# Latent profile analysis and associated factors of alexithymia among patients with chronic obstructive pulmonary disease

**DOI:** 10.3389/fpsyt.2026.1823787

**Published:** 2026-07-10

**Authors:** Liang Ji, Jing Xu, Xiao Huang, Shuxiang Chen, Pengmei Lin, Ziyue Chen, Qilin Shen, Fei Wang

**Affiliations:** 1 Mianyang Hospital Affiliated to Chengdu University of Traditional Chinese Medicine, Mianyang Hospital of Traditional Chinese Medicine (TCM), Mianyang, Sichuan, China; 2 Chengdu University of Traditional Chinese Medicine, Chengdu, Sichuan, China

**Keywords:** alexithymia, chronic obstructive pulmonary disease, latent profile analysis, mental health, older patient

## Abstract

**Background:**

Alexithymia is one of the most common psychological characteristics observed in patients with chronic obstructive pulmonary disease (COPD) and is associated with poorer health outcomes. The negative impact of alexithymia on physical illness is reflected in more severe clinical symptoms and poorer quality of life, which adversely affect clinical outcomes and further increase disease burden, potentially contributing to a cycle of worsening health status. Therefore, timely identification of the psychological characteristics of patients with COPD, along with early psychological support, may help improve the quality of life of patients with COPD.

**Objectives:**

To explore the latent profiles of alexithymia among patients with COPD and to analyze their associated factors.

**Methods:**

This cross-sectional study recruited 312 patients with COPD using convenience sampling between October 2024 and March 2025. Sociodemographic characteristics, physiological and disease-related information, psychospiritual information, environmental factors, and alexithymia scores were collected. Latent profile analysis, univariate analysis, and multinomial logistic regression were conducted to identify alexithymia profiles and examine factors associated with profile membership.

**Results:**

The results of this study showed that patients with COPD were categorized into three alexithymia profiles: high (28.85%), moderate (63.46%), and low (7.69%). COPD duration, depression, activity intensity, self-perceived social support, psychological resilience, and anxiety were associated with different alexithymia profiles among patients with COPD.

**Conclusion:**

This single-center cross-sectional study identified three preliminary alexithymia profiles (high, moderate, and low) among patients with COPD. Due to potential selection bias resulting from convenience sampling, these findings require validation in multicenter studies before generalization. The observed associations among COPD duration, depression, activity intensity, social support, resilience, and anxiety provide empirical evidence to inform the development of tailored patient classification and targeted support strategies for early identification, with the goal of improving quality of life in this population.

## 1 Introduction

Chronic obstructive pulmonary disease (COPD) is a chronic respiratory disease characterized by persistent airflow limitation and progressive deterioration. It is associated with high morbidity, prolonged disease duration, and multiple comorbidities and has become one of the leading causes of mortality worldwide. (1) Approximately 3.3 million people die from COPD each year globally, (2) and in China, COPD-related deaths are projected to reach 1,055,400 by 2030, with a mortality rate of 73.85 per 100,000 population. (3) Although COPD is primarily linked to exposure to harmful particles or gases, several intrinsic host factors, such as genetic susceptibility, abnormal inflammatory responses, and impaired lung development, also contribute to its pathogenesis. These complex mechanisms give rise to substantial heterogeneity in clinical manifestations (1).

Health anxiety is characterized by distressing emotional responses, such as fear, maladaptive cognitions, and heightened physiological arousal (4) Disease fluctuations, recurrent respiratory infections, and acute exacerbations often trigger fear and apprehension regarding disease progression among patients with COPD. (5, 6) Anxiety and depression are recognized as two of the eight most prevalent high-risk comorbidities in individuals with COPD. (1) Previous studies (7) have shown that alexithymia explains 14.6%–16.4% of the variance in depression and anxiety symptoms, with difficulty recognizing emotions identified as the strongest predictor across symptom domains. Consequently, alexithymia may represent an important psychological concern among patients with COPD.

Alexithymia refers to a cognitive–affective difficulty characterized by impairments in emotion recognition, processing, and regulation (8). As a result, individuals with alexithymia often struggle to accurately distinguish between bodily sensations and emotional states and have difficulty articulating their internal feelings or expressing negative emotions. These difficulties may complicate clinical assessment and are associated with poorer treatment-related outcomes and prognosis. (9) Current literature generally categorizes the pathogenesis of alexithymia into three major mechanisms. The first is the cognitive mechanism, characterized by deficits in emotional schemas, theory of mind, and executive functioning. The second is the neurological mechanism, in which structural and functional abnormalities across brain regions contribute to the development of alexithymia traits. The third involves sociopsychological factors, including adverse life experiences (e.g., childhood adversity, illness-related stress), sociocultural influences, and interpersonal dynamics. (10) Among these, psychosocial factors have garnered particular attention due to their modifiability and responsiveness to intervention. Most existing studies have explored the determinants of alexithymia in various patient populations from demographic, psychological, disease-related, familial, and social environmental perspectives. (11) However, these studies have largely been grounded in clinical experience rather than supported by a robust and systematic theoretical framework.

In 2010, Brant et al. (12) proposed the Dynamic Symptoms Model (DSM) as a theoretical framework for symptom management, emphasizing that symptoms are dynamic phenomena influenced by multiple factors, including physiological, psychological, environmental, and individual adaptive strategies. In 2016, the model was further revised to provide a more comprehensive depiction of symptom experiences, antecedents, and consequences, as well as the pathways through which interventions influence. The updated model helps clarify symptom influences in clinical practice and facilitates the development of research questions and analytical strategies. The DSM has been applied in research on symptom management across chronic diseases, mental health conditions, and acute illnesses, with particular focus on symptom typologies, trajectories, and intervention strategies for individuals with chronic conditions (13). In the present study, the DSM served as the theoretical framework to guide the systematic identification of potential determinants of alexithymia among patients with COPD. Influencing factors were explored comprehensively across four dimensions: sociodemographic characteristics, physiological and disease-related factors, psychological and spiritual factors, and environmental contexts. Existing evidence suggests that psychological problems in older adults with alexithymia warrant priority attention and targeted intervention by healthcare professionals compared with those in older adults without alexithymia. (14) However, substantial heterogeneity exists in the manifestations of alexithymia, and the boundaries of its assessment and diagnosis remain insufficiently defined.

The National Institute of Nursing Research advocates the integration of precision health approaches into chronic disease and symptom self-management to address major health challenges and support research efforts aimed at developing personalized strategies for symptom prevention and management across diverse populations and care settings. (15) In alignment with the Healthy China 2030 initiative, it is particularly important for healthcare providers to comprehensively analyze the complexity of chronic disease symptoms and to implement precision nursing interventions in a systematic manner.

Latent profile analysis (LPA) is a person-centered classification method that identifies homogeneous subgroups based on individual characteristics and thereby provides an empirical foundation for precision interventions. (16) LPA is grounded in maximum likelihood estimation and uses latent variables to explain the relationships among observed continuous indicators. This approach classifies individuals into a limited number of mutually exclusive latent categories with distinct profiles, ensuring local independence while minimizing within-class variability and maximizing between-class differentiation. Its classification accuracy and validity are objectively evaluated using model-fit indices. (16) To gain a deeper understanding of the specific manifestations of alexithymia among patients with COPD, a key objective of this study was to identify distinct alexithymia subtypes using LPA. This approach may assist clinicians in clarifying the typological differences in alexithymia among patients with COPD and, guided by the DSM framework, may help identify characteristic influencing factors associated with each latent class. Such findings may inform the development of targeted intervention strategies for patients with COPD and alexithymia while contributing to the construction of accurate patient profiles. This may facilitate the optimization of precision healthcare pathways for individuals with COPD.

## 2 Methodology

### 2.1 Design and procedure

The present study employed a cross-sectional design to assess the current status of alexithymia and to conduct LPA among patients with COPD. The study was conducted at a tertiary hospital in Sichuan, China, where clinical nurses recruited participants through face-to-face enrollment between October 2024 and March 2025. Prior to recruitment, the purpose of the study, survey content, and procedures were thoroughly explained to all potential participants, who were informed of their right to withdraw at any time. The study protocol was approved by the Research Ethics Committee of Mianyang Hospital Affiliated with Chengdu University of Traditional Chinese Medicine (approval number: 2021KL-04) and was conducted in accordance with the Declaration of Helsinki.

### 2.2 Participants

The inclusion criteria were as follows: (1) meeting the diagnostic criteria outlined in the Guidelines for the Diagnosis and Treatment of Chronic Obstructive Pulmonary Disease (2021 Revision) in China; (2) age ≥18 years; (3) clear consciousness and intact verbal communication ability; and (4) provision of informed consent and voluntary agreement to participate in the study. The exclusion criteria were as follows: (1) diagnosed mental illness or cognitive impairment; (2) presence of other severe organ diseases or malignant tumors; and (3) transfer to another hospital or clinical department during the study period.

During the recruitment period from October 2024 to March 2025, 322 eligible patients were approached. Among them, 312 agreed to participate and completed the survey, yielding a response rate of 96.89%. Nonparticipation among the 10 eligible non-consenters was due to lack of interest (n = 6) or time constraints (n = 4). Several potential sources of selection bias should be noted. First, recruitment from a single urban tertiary hospital may have overrepresented patients with greater illness severity or better access to specialized care while underrepresenting those with milder disease managed in community or primary care settings. Second, the online survey mode may have introduced digital access bias, as older, less technologically literate, or more severely ill patients with COPD may have been less likely to participate or complete the survey. Third, the convenience sampling approach—approaching available patients rather than using random sampling—may limit the representativeness of the sample. These biases are discussed further in the Limitations section.

### 2.3 Materials

This study used the DSM (12) as the theoretical framework to guide the comprehensive inclusion of factors influencing alexithymia characteristics among patients with COPD. Data were collected online using the Health-Related Behaviors Questionnaire for COPD Patients, which was developed by the research team. Only questions and scales relevant to the research objectives and hypotheses were included in this study. The questionnaire consisted of four sections: nine sociodemographic variables, seven physiological and disease-related variables, seven psychosocial-spiritual variables, and three environmental variables.

#### 2.3.1 Sociodemographic variables

Sociodemographic variables were collected through self-report surveys, including gender, age, marital status (married, divorced, widowed, or unmarried), education level (junior high school or below, senior high school, or college and above), living environment (urban or rural), living arrangement (living alone or living with relatives), occupational status (employed, retired, or unemployed), per capita monthly household income (insufficient to cover expenses or balanced income and expenses), and medical payment type (medical insurance or New Rural Cooperative Medical Scheme).

#### 2.3.2 Physiological and disease-related variables

Body mass index, duration of COPD (years), and family history of COPD (yes or no) were recorded. The Barthel Index (BI) (17) was used to assess patients’ self-care ability. The modified British Medical Research Council Dyspnea Scale (mMRC) (18) was used to evaluate dyspnea severity. This scale consists of five levels (0–4), with scores ranging from 0 to 4 to represent increasing degrees of dyspnea; higher scores indicate more severe dyspnea. Comprehensive symptom burden was assessed using the COPD Assessment Test (19), which consists of eight items, each scored from 0 to 5, yielding a total score ranging from 0 to 40; higher scores indicate greater symptom severity. The Tilburg Frailty Indicator (TFI) (20) was used to assess physical, psychological, and social frailty among patients with COPD. The total TFI score ranges from 0 to 15, with higher scores indicating greater frailty. The overall Cronbach’s α was 0.710, and the test–retest reliability was 0.880, indicating acceptable internal consistency and good stability.

#### 2.3.3 Psychosocial-spiritual variables

##### 2.3.3.1 Distress thermometer

The DT was proposed by Roth et al. (21) and translated into Chinese by Tang et al. (9) in 2010. The scale assesses the level of psychological distress in patients with COPD. It consists of 11 points ranging from 0 to 10, with higher scores indicating greater psychological distress. The cutoff score was 4, with an area under the curve of 0.80 and a test–retest reliability of 0.800 (*P* < 0.001).

##### 2.3.3.2 Connor–Davidson Resilience Scale

The CD-RISC was derived from (22) the original 25-item resilience scale developed by Campbell-Sills and Stein (22) and is used to measure psychological resilience in patients. The simplified version is a one-dimensional scale. It uses a 5-point Likert scale, yielding a total score ranging from 0 to 40, with higher scores indicating greater psychological resilience.

##### 2.3.3.3 Patient Health Questionnaire-2 and Generalized Anxiety Disorder-2

The PHQ-2 and GAD-2 were used to measure depressive and anxiety symptoms in patients with COPD. The PHQ-2 consists of the first two items of the Patient Health Questionnaire-9 (PHQ-9) (23), which represent the two core criteria for depressive disorders (24): (1) depressed mood, feelings of depression, or hopelessness; and (2) diminished interest or pleasure in activities. The total score ranges from 0 to 6, and a cutoff score of ≥3 suggests the presence of clinically relevant depressive symptoms. The GAD-2 consists of the first two items of the Generalized Anxiety Disorder-7 (GAD-7) (25) and reflects the two core criteria for anxiety disorders (26): (1) feeling nervous, anxious, or on edge; and (2) inability to stop or control worrying. The total score ranges from 0 to 6, and a cutoff score of ≥3 indicates clinically relevant anxiety symptoms. In this study, Cronbach’s α values for the PHQ-2 and GAD-2 were 0.812 and 0.831, respectively. The PHQ-2 and GAD-2 are ultra-brief screening instruments rather than diagnostic tools. They are designed for initial risk stratification rather than precise measurement of depression or anxiety severity. In this study, their sum scores were treated as continuous predictors for analytical consistency; however, this approach assumes interval-level measurement properties that these ordinal scales may not fully satisfy.

##### 2.3.3.4 Emotion Regulation Questionnaire

The ERQ was developed by Gross and John (27). It was used to assess participants’ habitual use of emotion regulation strategies. The scale consists of 10 items across two dimensions: expressive suppression (4 items) and cognitive reappraisal (6 items). Each item is rated on a 7-point Likert scale, yielding a total score ranging from 10 to 70. Higher mean scores for each dimension indicate more frequent use of the corresponding regulation strategy. In this study, Cronbach’s α coefficients for expressive suppression and cognitive reappraisal were 0.808 and 0.846, respectively.

##### 2.3.3.5 Perceived Social Support Scale

The PSSS was developed by Zimet et al. (28) to assess perceived social support among study participants. The scale consists of 12 items across three dimensions: family support (items 11, 3, 4, and 8), friend support (items 6, 7, 9, and 12), and other support (items 1, 2, 5, and 10). Items are rated on a 7-point Likert scale ranging from 1 (“strongly disagree”) to 7 (“strongly agree”), yielding total scores from 12 to 84. Higher scores indicate greater perceived social support. In this study, Cronbach’s α for the PSSS was 0.845.

##### 2.3.3.6 Big Five Personality Inventory—Neuroticism

A validated 44-item measure of the Big Five personality traits was used (29) Participants indicated their agreement with each statement on a 5-point Likert scale ranging from 1 (“strongly disagree”) to 5 (“strongly agree”). The inventory evaluates five domains: extraversion, openness, conscientiousness, neuroticism, and agreeableness. Eight items assess neuroticism (e.g., “is depressed, blue”). In this study, Cronbach’s α for the neuroticism subscale was 0.886.

#### 2.3.4 Environmental variables

(1) Smoking status (never smoked, current smoker, or former smoker); (2) drinking status (never drank, current drinker, or former drinker); and (3) International Physical Activity Questionnaire (IPAQ). The IPAQ was developed by Craig et al. (30) and revised in Chinese by Qu and Li (31). It includes domains related to work, transportation, household activities, leisure-time physical activity, and sedentary behavior. Respondents were asked to recall, as accurately as possible, their physical activities related to work, transportation, and leisure time during the past 7 days. The Metabolic Equivalent of Task (MET) was used as an indicator to evaluate the relative energy expenditure of physical activity. One MET represents an exercise intensity corresponding to 3.5 mL of oxygen consumed per kilogram of body weight per minute of activity. Activities were classified into three levels based on MET values: light (<3.0 METs), moderate (3.0–6.0 METs), and vigorous (>6.0 METs). Data were assigned and calculated according to the following definitions: (1) total MET for walking = 3.3 × (work + transportation + leisure time on foot); (2) total MET for moderate-intensity activities = 6.0 × transportation time by bicycle + 4.0 × (outdoor household moderate-intensity activities + work-related moderate-intensity activities + leisure-time moderate-intensity activities) + 3.0 × indoor household moderate-intensity activities + 5.5 × household vigorous-intensity activities; and (3) total MET for high-intensity activities = 8.0 × (work-related vigorous-intensity activities + leisure-time vigorous-intensity activities) (32, 33). Total physical activity was calculated as MET-minutes per week following the IPAQ scoring protocol (30): Walking MET-minutes/week = 3.3 × (walking minutes/day) × (walking days/week); Moderate-intensity MET-minutes/week = 4.0 × (moderate activity minutes/day) × (moderate activity days/week); Vigorous-intensity MET-minutes/week = 8.0 × (vigorous activity minutes/day) × (vigorous activity days/week). Total MET-minutes/week was calculated as the sum of these three components. Based on total MET-minutes per week, participants were classified into three activity levels according to IPAQ guidelines (30): low activity level (<600 MET-minutes/week), moderate activity level (600 to <3000 MET-minutes/week), and high activity level (≥3000 MET-minutes/week).

#### 2.3.5 Outcome measurement

##### 2.3.5.1 Toronto Alexithymia Scale-20

The TAS-20 was developed by Bagby et al. (34) in 1994 and translated into Chinese by Yi et al. (35). The scale is widely used to assess alexithymic traits and to measure the level of alexithymia in both clinical and general populations. It comprises three dimensions: difficulty identifying feelings (7 items), difficulty describing feelings (5 items), and externally oriented thinking (8 items). The instrument contains 20 items rated on a 5-point Likert scale, with each item scored from 1 (“strongly disagree”) to 5 (“strongly agree”). The total score ranges from 20 to 100, with higher scores indicating greater alexithymia. In this study, Cronbach’s α for the scale was 0.810.

### 2.4 Statistical analysis

There were no missing data in the final dataset of 312 participants because the online survey platform required responses to all items before submission. Therefore, no imputation methods were applied. No adjustment for multiple comparisons (e.g., Bonferroni correction) was performed because the analyses were primarily exploratory in nature; thus, the results should be interpreted with caution. First, LPA (36) was conducted using Mplus version 8*.3*. Model fit was evaluated using the Akaike Information Criterion (AIC), Bayesian Information Criterion (BIC), adjusted BIC (aBIC), entropy, the Lo–Mendell–Rubin likelihood ratio test (LMRT), and the bootstrap likelihood ratio test (BLRT). Smaller values of AIC, BIC, and aBIC indicate better model fit (37). Entropy values greater than 0.80 indicate acceptable classification accuracy, with values closer to 1.00 reflecting higher precision (38). Statistically significant LMRT and BLRT results (P < 0.05) indicate that the LPA model with *k* profiles fits significantly better than the model with *k* − *1* profiles, whereas nonsignificant p-values suggest that the model with *k* − *1* profiles provides a more parsimonious fit (36, 39). The number of participants in each profile was required to exceed 5% of the total sample (39). In addition, the interpretability and substantive significance of the identified profiles were considered when determining the optimal number of profiles. (40, 41) Second, all variables were assessed for multicollinearity using *IBM SPSS* Statistics version 25. Descriptive statistics were calculated for variables across profiles, and one-way analysis of variance (ANOVA) was performed to examine between-group differences. Means and standard deviations were used to describe normally distributed continuous variables, and comparisons between groups were performed using independent-samples t tests or one-way ANOVA. Medians and interquartile ranges were used to describe non-normally distributed continuous variables, and comparisons between groups were conducted using *the Mann*–*Whitney U* test or *the Kruskal*–*Wallis H* test. Frequencies and percentages were used to describe categorical variables, and the chi-square test was used for between-group comparisons. Statistical significance was set at *P* < 0.05. Finally, variables that were statistically significant in the univariate analyses were entered into a multinomial logistic regression model to examine independent factors associated with alexithymia profile membership among patients with COPD.

## 3 Results

### 3.1 Participant characteristics and model selection

The online survey system recorded 312 valid responses. Detailed demographic and clinical characteristics are presented in **Table 1**. Participants demonstrated relatively high levels of alexithymia (M = 59.11, SD = 9.01), difficulty identifying feelings (*M* = 20.43, *SD* = 4.44), difficulty describing feelings (*M* = 14.58, *SD* = 2.69), and externally oriented thinking (*M* = 24.10, *SD* = 3.21). The three subscale scores of the Toronto Alexithymia Scale—difficulty identifying feelings, difficulty describing feelings, and externally oriented thinking—were used as indicators in the LPA to identify latent alexithymia profiles among patients with COPD. Using a stepwise approach, we began with an unconditional one-profile model and subsequently estimated models with two to five profiles. As the number of profiles increased, the log-likelihood (LL) values increased, AIC, BIC, and aBIC values decreased, entropy improved, and the BLRT remained statistically significant. Significant BLRT and LMRT values were observed up to the five-profile model. Fit indices for the competing latent profile models are presented in **Table 2**.

**Table 1:** Demographic and clinical characteristics (*N* = 312).

Variables		N (%)/M (P_25_, P_75_)	Variables		N (%)/M (P_25_, P_75_)
Sociodemographic variables					
Gender	Men	168(53.8)	Occupational status	Be employed	25(8.0)
	Women	144(46.2)		Retirement	274(87.8)
Marital status	Married	236(75.6)		Separation	13(4.2)
	Divorced/Widowed/Unmarried	76(24.4)	Type of monthly per capita household income	Excess of income over expenditure	49(15.7)
Age		70.00(62.25, 74.00)		Balance of income over expenditure	97(31.1)
Living environment	Urban	223(71.5)		Excess of income over expenditure	166(53.2)
	Rural	89(28.5)	Medical payment types	Medical insurance	191(61.2)
Educational level	Junior high school and below	166(53.2)		New rural cooperative medical care	121(38.8)
	High school or junior college	105(33.7)	Residency Style	Living alone	63(20.2)
	College and above	41(13.1)		Living with relatives	249(79.8)
Physiological disease variables					
BMI		24.00(21.60,25.50)	ERQ		44.00(38.00,50.00)
COPD Duration(year)		9.00(5.25,15.00)	PSSS		44.00(36.00,53.00)
BI		75.00(60.00,85.00)	BFI-N-O		24.00(21.00,26.00)
mMRC		2.00(1.00,3.00)	Environment variable		
CAT		16.50(10.00,25.00)	Smoking status	Never smoked	144(46.2)
Family history of COPD	Yes	147(47.1)		Smoked	90(28.8)
	No	165(52.9)		Quit smoking	78(25.0)
TFI		7.00(5.00,9.00)	Alcohol status	Never drank	136(43.6)
Psycho-spiritual variables				Drank	89(28.5)
DT		5.00(3.00,7.00)		Quit drinking	87(27.9)
CD-RISC10		20.00(17.00,27.00)	Level of activity	Low-intensity activity	99(31.7)
PHQ-2		2.00(1.00,3.00)		Moderate intensity activities	131(42.0)
GAD-2		2.00(1.00,3.00)		High intensity activity	82(26.3)

BI, Barthel index; mMRC, modified British medical research council; CAT, COPD assessment test; TFI, Tilburg frailty indicator; DT, distress thermometer; CD-RISC10, Connor Davidson resilience; ERQ, emotion regulation; PSSS, perceived social support; BFI-N-O, bigfive.

**Table 2:** Fit Statistics for profile structures (*N* = 312).

Number of profiles	LL	AIC	BIC	aBIC	*p*LMR	*p*BLRT	Entropy	Group size for each profile				
								1	2	3	4	5
1	-2464.233	4940.465	4962.923	4943.893	_	_	_	312(100.00%)				
2	-2349.399	4718.798	4756.229	4724.512	<0.0000	<0.0000	0.752	114(36.57%)	198(63.43%)			
3	-2293.941	4615.882	4668.284	4623.881	0.0005	<0.0000	0.842	90(28.85%)	198(63.46%)	24(7.69%)		
4	-2269.085	4574.170	4641.544	4584.454	0.2547	<0.0000	0.861	7 (2.24%)	100(32.06)	23(7.37%)	182(58.33%)	
5	-2253.698	4551.396	4633.742	4563.966	0.1912	<0.0000	0.810	7(2.24%)	80(25.64%)	141(45.19%)	69(22.12%)	15(4.81%)

LL, log-likelihood; FP, free parameters; AlC, Akaike information criteria; BlC, Bayesian information criteria; sBlC, sample-size- adjusted BIC; pLMR, p-value for LoMendell-Rubin adjusted likelihood ratio test for K vs. K-1 profiles; pBLRT, p-value for Bootstrapped Likelihood Ratio Test. The numbers 1–5 in the rightmost columns indicate profile numbers. Values in parentheses are percentages of the total sample (*N* = 312).

Although the four-profile and five-profile solutions yielded lower LL, AIC, BIC, and aBIC values than the three-profile solution, their LMRT results were not statistically significant, suggesting that these models did not provide a meaningful improvement over models with one fewer profile. In addition, both the four-profile and five-profile solutions generated very small classes, with at least one profile comprising less than 5% of the total sample, which may compromise classification stability and interpretability. Therefore, these solutions were not retained. The three-profile solution demonstrated acceptable model fit, with significant LMRT and BLRT results, entropy greater than 0.80, no profile smaller than 5% of the sample, and clear clinical interpretability. Accordingly, the three-profile solution was selected as the final model.

### 3.2 Profile characteristics

**Figure 1:**
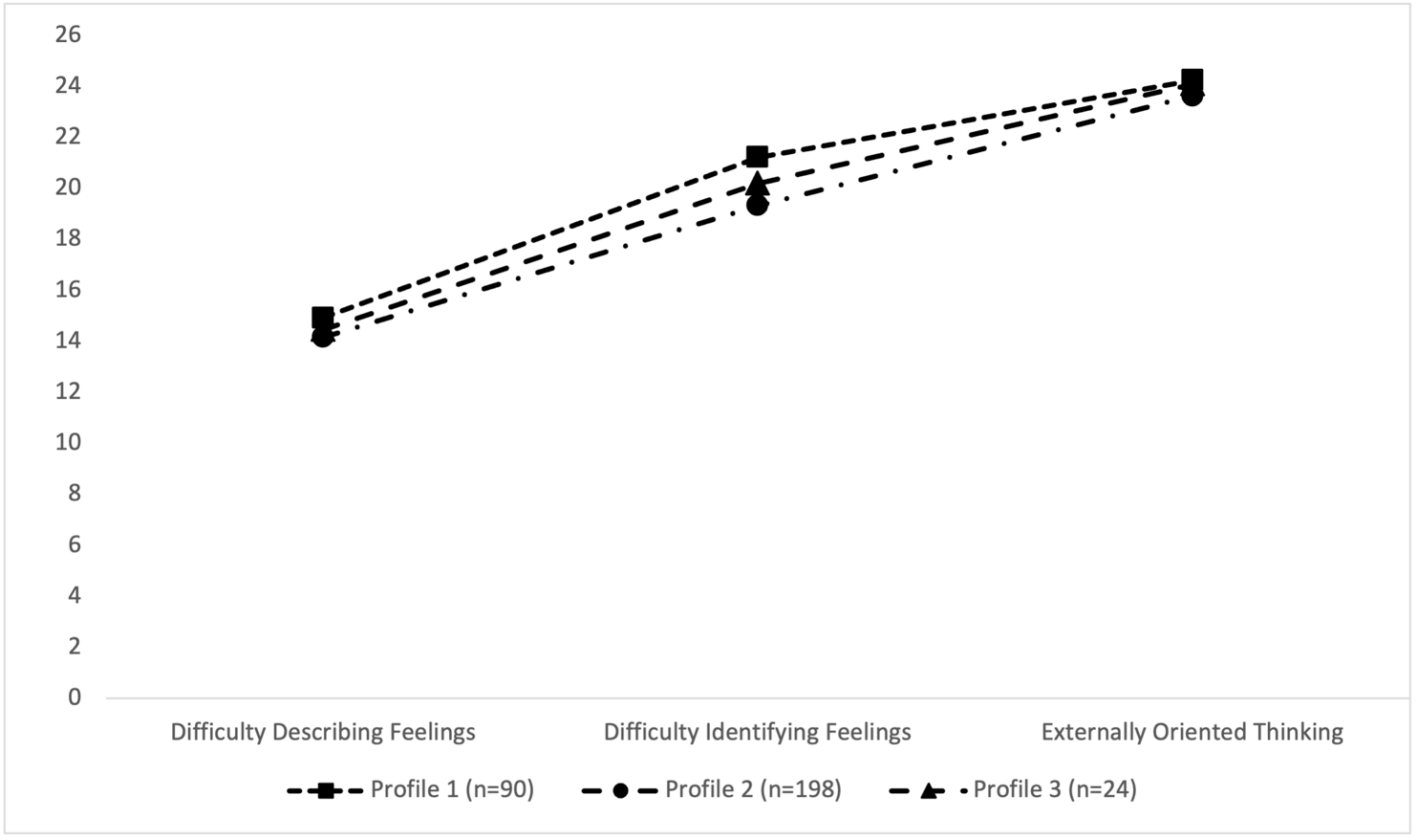
Mean scores of TAS-20 dimensions across the three latent profiles: Profile 1 = high alexithymia group, Profile 2 = moderate alexithymia group, and Profile 3 = low alexithymia group.

Based on the final three-profile solution, the latent profiles were named according to the mean scores of the three TAS-20 dimensions. Profile 1 was labeled the high alexithymia group, Profile 2 the moderate alexithymia group, and Profile 3 the low alexithymia group. As shown in **Figure 1**, the horizontal axis represents the three dimensions of alexithymia, and the vertical axis indicates the corresponding scores. Profile 1, classified as the high alexithymia group, exhibited the highest levels of difficulty identifying feelings (*M* = 21.33, *SD* = 4.48), difficulty describing feelings (*M* = 14.93, *SD* = 2.50), and externally oriented thinking (*M* = 24.24, *SD* = 2.98), accounting for 28.85% of the sample. Profile 2 demonstrated moderate levels of difficulty identifying feelings (*M* = 20.20, *SD* = 4.34), difficulty describing feelings (*M* = 14.46, *SD* = 2.74), and externally oriented thinking (*M* = 24.10, *SD* = 3.29), accounting for 63.46% of the sample. Profile 3 showed the lowest levels of difficulty identifying feelings (*M* = 19.33, *SD* = 4.78), difficulty describing feelings (*M* = 14.17, *SD* = 2.91), and externally oriented thinking (*M* = 23.63, *SD* = 3.47), accounting for 7.69% of the sample, and was labeled the low alexithymia group.

### 3.3 Predictors of latent alexithymia profiles

As shown in **Table 3**, all variance inflation factor (VIF) values were below 5 (ranging from 1.209 to 4.585), and tolerance values were greater than 0.100, indicating no evidence of severe multicollinearity. Although GAD-2 (4.585), PHQ-2 (4.530), CD-RISC-10 (3.804), and DT (3.421) demonstrated moderate collinearity, these values remained within acceptable thresholds. Therefore, all variables were retained to preserve theoretical coherence guided by the DSM framework. Variables that were statistically significant (P < 0.050) in the univariate analyses were entered into the multinomial logistic regression model as predictors. However, occupational status was excluded from the final model because sparse cells in Profile 3 produced unstable parameter estimates, suggestive of quasi-complete separation. Consequently, the final model included age, living arrangement, COPD duration, BI, mMRC, DT, CD-RISC-10, PHQ-2, GAD-2, ERQ, PSSS, BFPI-N, smoking status, alcohol consumption status, and physical activity level.

**Table 3:** Comparison of the characteristics of statistical variables between different profiles.

Variables		Profile 1: High alexithymia group (n = 90) [N/%, M/*95%CI*]	Profile 2: Moderate alexithymia group (n = 198) [N/%, M/*95%CI*]	Profile 3: Low alexithymia group (n = 24) [N/%, M/*95%CI*]	Statistical value	*P* value	Collinearity diagnostics	
							Tolerance	*VIF*
Sociodemographic variables								
Gender	Men	56(62.2)	97(49.0)	15(62.5)	5.143^a^	0.076	0.383	2.611
	Women	34(37.8)	101(51.0)	9(37.5)				
Age	68.55(67.49,69.61)	71.32(69.03,73.62)	67.06(65.82,68.29)	70.46(67.70,73.21)	7.005	0.001	0.494	2.024
Marital status	Married	63(70.0)	154(77.8)	19(79.2)	2.207^a^	0.332	0.685	1.460
	Divorced/Widowed/Unmarried	27(30.0)	44(22.2)	5(20.8)				
Educational level	Junior high school and below	47(52.2)	108(54.5)	11(45.8)	2.032^a^	0.730	0.654	1.528
	High school or junior college	30(33.3)	64(32.3)	11(45.8)				
	College and above	13(14.4)	26(13.1)	2(8.4)				
Living environment	Urban	60(66.7)	144(72.7)	19(79.2)	1.869^a^	0.393	0.474	2.109
	Rural	30(33.3)	54(27.3)	5(20.8)				
Residency Style	Living alone	13(14.4)	39(19.7)	11(45.8)	11.667^a^	0.003	0.767	1.304
	Living with relatives	77(85.6)	159(80.3)	13(54.2)				
Occupational status	Be employed	3(3.3)	22(11.1)	_	11.689^a^	0.020	0.733	1.364
	Retirement	80(88.9)	170(85.9)	24(100)				
	Separation	7(7.8)	6(3.0)	_				
Type of monthly percapita household income	Excess of income over expenditure	19(21.1)	27(13.6)	3(12.5)	3.107^a^	0.540	0.448	2.23
	Balance of income over expenditure	25(27.8)	65(32.8)	7(29.2)				
	Excess of income over expenditure	46(51.1)	106(53.5)	14(58.3)				
Medical payment types	Medical insurance	48(53.3)	127(64.1)	16(66.7)	3.370^a^	0.185	0.446	2.243
	New rural cooperative medical care	42(46.7)	71(35.9)	8(33.3)				
Physiological disease variables								
BMI	23.93(23.52,24.33)	23.30(22.74,23.84)	24.29(23.74,24.84)	23.34(21.53,25.14)	2.675^b^	0.070	0.805	1.241
COPD Duration(year)	10.75(9.95,11.54)	13.21(11.65,14.78)	9.41(8.47,10.35)	12.54(9.74,15.35)	10.140^b^	<0.001	0.586	1.706
Family history of COPD	Yes	43(47.8)	88(44.4)	16(66.7)	4.265^a^	0.119	0.827	1.209
	No	47(52.2)	110(55.6)	8(33.3)				
BI	71.33(69.36,73.30)	65.62(61.44,69.81)	74.66(72.46,76.86)	65.21(57.70,72.72)	10.209^b^	<0.001	0.362	2.76
mMRC	1.81(1.68,1.95)	3.27(3.03,3.51)	2.54(2.37,2.71)	3.38(2.90,3.85)	14.789^b^	<0.001	0.216	4.625
CAT	17.99(16.90,16.98)	17.56(15.76,19.35)	18.27(16.84,19.70)	17.29(12.66,21.92)	0.288^b^	0.796	0.514	1.947
TFI	6.71(6.29,7.12)	7.27(6.60,7.93)	6.71(6.29,7.12)	7.54(6.24,8.84)	1.574^b^	0.209	0.752	1.33
Psycho-spiritual variables								
DT	4.33(4.03,4.63)	4.88(4.37,5.38)	4.05(3.66,4.43)	4.62(3.37,5.87)	2.371^b^	0.010	0.292	3.421
CD-RISC10	21.14(20.26,22.02)	23.33(21.68,24.98)	19.74(18.66,20.83)	24.42(21.44,27.38)	9.035^b^	<0.001	0.263	3.804
PHQ-2	2.06(1.88,2.25)	1.57(1.23,1.90)	2.31(2.08,2.54)	1.88(1.14,2.61)	6.586^b^	0.002	0.221	4.530
GAD-2	2.13(1.94,2.32)	1.31(0.99,1.63)	2.54(2.31,2.77)	1.83(1.10,2.57)	18.838^b^	<0.001	0.218	4.585
ERQ	44.33(43.29,45.37)	45.83(44.20,47.47)	43.14(41.77,44.51)	48.58(44.71,52.46)	5.423^b^	0.005	0.675	1.482
PSSS	44.25(43.07,45.43)	51.11(49.19,53.03)	40.61(39.24,41.97)	48.58(45.03,52.14)	40.638^b^	<0.001	0.365	2.736
BFI-N-O	24.27(23.64,24.90)	22.76(21.57, 23.94)	24.95(24.17,25.73)	24.38(22.15,26.60)	4.786^b^	0.009	0.371	2.696
Environment variable								
Smoking status	Never smoked	33(36.7)	103(52.0)	8(33.3)	45.387^a^	<0.001	0.245	4.089
	Smoked	16(17.7)	70(35.4)	4(16.7)				
	Quit smoking	41(45.6)	25(12.6)	12(50.0)				
Alcohol status	Never drank	27(30.0)	101(51.0)	8(33.3)	41.247^a^	<0.001	0.278	3.596
	Drank	20(22.2)	66(33.3)	3(12.5)				
	Quit drinking	43(47.8)	31(15.7)	13(54.2)				
Level of activity	Low-intensity activity	23(25.6)	68(34.3)	8(33.3)	18.901^a^	0.001	0.716	1.397
	Moderate intensity activities	52(57.8)	66(33.4)	13(54.2)				
	High intensity activity	15(16.7)	64(32.3)	3(12.5)				

a is X^2^value; b is F value; BI, Barthel index; mMRC, modified British medical research council; CAT, COPD assessment test; TFI, Tilburg frailty indicator; DT, distress thermometer; CD-RISC10, Connor Davidson resilience; ERQ, emotion regulation; PSSS, perceived social support; BFI-N-O, big five; TAS, Alexithymia.

The factors independently associated with alexithymia profiles among patients with COPD were examined using multinomial logistic regression, with the high alexithymia group as the reference category. Variables included in the final model were selected based on the results of the univariate analyses and model stability considerations. **Table 4** presents the results of the multinomial logistic regression comparing different profiles. Specifically, longer COPD duration was associated with significantly lower odds of belonging to the moderate alexithymia group compared with the high alexithymia group (OR = 0.923, 95% CI: 0.869–0.981), indicating that patients with longer disease duration were more likely to belong to the high alexithymia group. Higher depression scores (PHQ-2) were associated with lower odds of membership in the moderate alexithymia group (OR = 0.557, 95% CI: 0.340–0.912), suggesting that more severe depressive symptoms were linked to the high alexithymia group. Similarly, higher anxiety scores (GAD-2) were associated with lower odds of being in the moderate alexithymia group (OR = 0.852, 95% CI: 0.806–0.901), indicating that greater anxiety was related to an increased likelihood of belonging to the high alexithymia group. Compared with patients engaging in high-intensity physical activity, those with low-intensity activity (OR = 0.308, 95% CI: 0.099–0.960) and moderate-intensity activity (OR = 0.208, 95% CI: 0.078–0.556) had significantly lower odds of belonging to the moderate alexithymia group, indicating that lower activity levels were associated with the high alexithymia profile. Lower levels of self-perceived social support (PSSS) were associated with higher odds of belonging to the moderate alexithymia group compared with the high alexithymia group (OR = 2.736, 95% CI: 1.640–4.654), indicating that poorer perceived social support was more strongly related to the high alexithymia profile. Higher psychological resilience (CD-RISC-10) was associated with higher odds of membership in both the moderate alexithymia group (OR = 1.093, 95% CI: 1.010–1.182) and the low alexithymia group (OR = 1.162, 95% CI: 1.028–1.314) relative to the high alexithymia group, suggesting that greater resilience was associated with a lower likelihood of high alexithymia. However, given the small size of the low alexithymia subgroup (n = 24), this finding should be interpreted as preliminary and requires replication. After applying the Benjamini–Hochberg false discovery rate (FDR) correction (FDR threshold = 0.05) for the 36 comparisons in the multinomial logistic regression model, GAD-2, PSSS, and moderate-intensity physical activity remained statistically significant (all P_FDR < 0.05). Other variables that showed associations in the unadjusted analyses, including COPD duration, CD-RISC-10, PHQ-2, and low-intensity physical activity, did not remain statistically significant after FDR correction (**Table 4**). The following results are interpreted in light of this distinction. To operationalize the DSM analytically, we tested the interaction between COPD duration and psychological resilience (CD-RISC-10) in a multinomial logistic regression model using the high alexithymia group (Profile 1) as the reference category. The interaction term was not statistically significant for either comparison (Profile 2 vs 1: B = 0.003, P = 0.588; Profile 3 vs 1: B = 0.006, P = 0.077), providing no robust evidence of moderation. Therefore, these exploratory interaction results are not discussed further.

**Table 4:** Multinomial logistic regression model variable results.

Variables	Profile 2: Moderate alexithymia group (n = 198) VS Profile 1: High alexithymia group (n = 90)							Profile 3: Low alexithymia group (n = 24) VS Profile 1: High alexithymia group (n = 90)						
	*B*	*SE*	*Exp(B)*	*95% CI*		*P -RAW*	*P -FDR*	*B*	*SE*	*Exp(B)*	*95% CI*		*P -RAW*	*P -FDR*
				Lower	Upper						Lower	Upper		
Intercept	0.821	3.004				0.785	_	-20.807	5.131				<0.001	_
Age	0.035	0.026	1.035	0.983	1.090	0.187	0.0208	-0.033	0.041	0.967	0.892	1.048	0.418	0.0319
Residency style														
Living alone(ref)	0	—	1											
Living with relatives	0.169	0.448	1.184	0.492	2.849	0.706	0.0444	1.523	0.593	4.585	1.435	14.645	0.010	0.0069
COPD Duration(year)	0.080	0.031	0.923	0.869	0.981	0.009	0.0056	-0.035	0.041	0.966	0.891	1.047	0.394	0.0292
BI	0.020	0.016	1.020	0.988	1.053	0.226	0.0222	-0.003	0.027	0.997	0.946	1.050	0.897	0.486
mMRC	0.325	0.305	0.722	0.397	1.314	0.287	0.0250	0.767	0.487	2.154	0.830	5.589	0.115	0.0167
DT	0.062	0.106	1.064	0.865	1.309	0.559	0.040	-0.276	0.177	0.759	0.536	1.075	0.120	0.018
CD-RISC10	0.088	0.040	1.093	1.010	1.182	0.028	0.011	0.150	0.063	1.162	1.028	1.314	0.016	0.008
PHQ-2	-0.586	0.252	0.557	0.340	0.912	0.020	0.010	-0.112	0.418	0.894	0.394	2.026	0.788	0.047
GAD-2	-0.160	0.028	0.852	0.806	0.901	<0.001	0.001	-0.071	0.043	0.931	0.855	1.014	0.101	0.014
ERQ	0.025	0.022	1.025	0.982	1.070	0.260	0.024	0.057	0.038	1.058	0.983	1.140	0.135	0.019
PSSS	1.016	0.266	2.763	1.640	4.654	<0.001	0.003	0.407	0.415	1.502	0.665	3.389	0.328	0.028
BFI-N-O	-0.020	0.046	0.980	0.896	1.073	0.665	0.026	0.070	0.070	1.073	0.936	1.230	0.314	0.043
Smoking status														
Quit smoking(ref)	0	—	1											
Never smoked	0.439	0.720	1.552	0.378	6.367	0.542	0.039	-1.595	2.202	0.203	0.003	15.21	0.469	0.036
Smoked	1.333	0.577	3.793	1.224	11.75	0.521	0.038	-0.274	0.861	0.761	0.141	4.109	0.751	0.046
*Alcohol status*														
Quit drinking(ref)	0	—	1											
Never drank	0.558	0.711	1.747	0.434	7.039	0.433	0.033	1.785	2.193	5.959	0.081	438.644	0.416	0.031
Drank	0.858	0.544	2.357	0.811	6.852	0.115	0.015	-0.059	0.865	0.943	0.173	5.135	0.946	0.050
Level of activity														
High intensity activity(ref)	0	—	1											
Low-intensity activity	-1.176	0.579	0.308	0.099	0.960	0.042	0.013	0.739	0.967	2.094	0.315	13.923	0.445	0.035
Moderate intensity activities	-1.570	0.502	0.208	0.078	0.556	0.002	0.004	0.368	0.839	1.445	0.279	7.475	0.661	0.042

Profile 1 serves as the control group; BI, Barthel index; mMRC, modified British medical research council; CAT, COPD assessment test; TFI, Tilburg frailty indicator; DT, distress thermometer; CD-RISC10, Connor Davidson resilience; ERQ, emotion regulation; PSSS, perceived social support; BFI-N-O, big five; TAS, Alexithymia. Due to thesmall sample size of Profile 3 (n=24, 7.69% of the total sample), the estimates for comparisons involving Profile 3 have wide confidence intervals and should be interpreted with caution. These findings require replication in larger samples.

## 4 Discussion

### 4.1 Identification of three alexithymia profiles

Before discussing the specific findings, it is important to contextualize them within the study’s sampling framework. Participants were recruited from a single urban tertiary hospital in Mianyang, China, using convenience sampling. This sampling strategy may introduce selection bias, as the sample may overrepresent patients with COPD who have greater symptom burden, higher healthcare-seeking behavior, or better digital literacy due to the online survey format. Conversely, patients with milder disease, those managed in community settings, or those with limited internet access may be underrepresented. Therefore, the latent profiles and associated factors identified in this study should be considered preliminary and specific to a tertiary-care, urban, predominantly older and retired population of patients with COPD. Generalizability to broader COPD populations requires confirmation in more diverse, multicenter studies.

This study aimed to identify latent profiles of alexithymia among patients with COPD. Previous research has predominantly adopted a variable-centered approach to examine alexithymia in this population. In contrast, the present study employed a person-centered approach and identified three distinct alexithymia profiles. These findings suggest that each profile demonstrates unique patterns of alexithymic characteristics. These profile-specific characteristics provide a scientific basis for healthcare professionals to accurately identify alexithymia patterns among patients with COPD, which may, in turn, facilitate individualized assessment and targeted nursing interventions.

In this study, alexithymia among patients with COPD was classified into three profiles—high alexithymia, moderate alexithymia, and low alexithymia—using LPA. These findings indicate significant heterogeneity in alexithymia within this population. The high alexithymia group comprised 28.85% of patients, with a mean total alexithymia score of *M* = 60.41 (*SD* = 8.54), representing the highest levels among the three profiles. Characterized by elevated scores across all three dimensions, individuals in this group exhibited pronounced alexithymia traits. They were often unable to accurately articulate specific feelings, even when they had some awareness of internal emotional states, and tended to attribute emotional distress to external events or somatic symptoms rather than describing their emotional experiences to others. The moderate alexithymia group included the largest proportion of patients (63.46%), with a mean alexithymia score of M = 58.76 (SD = 9.06). Individuals in this group demonstrated intermediate levels across the three dimensions. For patients in the high and moderate alexithymia groups, attention should be directed toward identifying risk factors and screening high-risk individuals. These patients should receive guidance in distinguishing emotional somatization from disease-related symptoms, as timely intervention may contribute to improved disease management and psychological well-being. The low alexithymia group accounted for 7.69% of patients, with a mean alexithymia score of M = 57.13 (SD = 10.09), and showed relatively low scores across all three dimensions. Patients in this group may have a lower risk of clinically significant alexithymia; therefore, preventive education is recommended, including information about alexithymia and instruction in psychological relaxation techniques.

### 4.2 Influencing factors of the three alexithymia profiles

The results indicated that patients with COPD who had a longer disease duration were more likely to belong to the high alexithymia group. COPD is a respiratory disorder characterized by persistent airflow limitation and progressive physiological decline. As disease duration increases, patients may experience more severe dyspnea and a greater number of comorbidities, which may impair cognitive and sensory functioning and increase vulnerability to alexithymia (42, 43). Furthermore, prolonged exposure to COPD may subject patients to sustained physical distress and recurrent treatment-related challenges. Over time, this cumulative burden may erode confidence in treatment and prognosis, foster negative emotions, and consequently increase the risk or severity of alexithymia (44). Therefore, healthcare professionals should assess patients’ psychological status in a timely manner according to disease duration, provide targeted health education and follow-up support, and encourage the adoption of adaptive coping strategies. Such measures may help prevent or mitigate the progression of alexithymic traits in individuals with COPD. However, this association did not remain statistically significant after FDR correction for multiple comparisons (P_FDR = 0.102); therefore, this finding should be interpreted as exploratory and requires replication in larger samples.

These findings suggest that depressive symptoms were associated with alexithymia profile membership among patients with COPD. Individuals with more severe depressive symptoms were more likely to be classified in the high alexithymia group. Among patients with COPD, the presence of depressive symptoms may be associated with reduced emotional expression. (43) Depression often diminishes emotional expressiveness and reduces the skills and motivation required for effective emotional communication (45). As a result, patients may experience varying degrees of affective impairment, accompanied by decreased responsiveness to external stimuli and lower motivation for interpersonal interaction. These changes may partly explain the observed association between depressive symptoms and alexithymia. Moreover, older adults with depression frequently exhibit distinct physiological and emotional reactions. Under the influence of persistent negative mood, a vicious cycle may develop in which emotional distress becomes increasingly difficult to regulate. Their capacity to accurately recognize, interpret, and articulate their own emotional experiences may gradually decline, ultimately exacerbating the severity of alexithymia traits (46). Nevertheless, after FDR correction, the association between PHQ-2 scores and alexithymia profiles did not remain statistically significant (P_FDR = 0.144). This finding may reflect the moderate multicollinearity between depressive and anxiety symptoms (PHQ-2 and GAD-2 were correlated; VIF = 4.530), suggesting that anxiety may represent a more prominent correlate in this sample. Future studies using more comprehensive diagnostic instruments (e.g., PHQ-9) are needed to disentangle the independent contributions of depressive and anxiety symptoms.

The associations of self-perceived social support and physical activity intensity with alexithymia profiles remained statistically significant after FDR correction, providing more robust evidence for these relationships. The results indicate that patients with COPD who reported low or moderate levels of physical activity were more likely to belong to the high alexithymia group. Previous research has shown that various forms of physical activity are associated with improvements in physiological function among older adults, enhancement of psychological well-being, and a slowing of age-related physical and mental decline (47). Comparisons of older adults with different levels of physical activity suggest that moderate engagement in exercise is associated with significant improvements in physical function. Enhanced physical capacity may promote vitality, enabling older adults to demonstrate greater initiative and participation in interpersonal communication and social activities. Increased social engagement and communication, facilitated by improved physical and emotional functioning, may, in turn, help mitigate alexithymia tendencies among older adults. The findings further indicate that patients with COPD who reported lower self-perceived social support were more likely to be classified in the high alexithymia group. Individuals with poor perceived social support may lack the initiative to seek available assistance from their social networks and may be less aware of care and support provided by family members or friends. This insufficient perception of support may increase vulnerability to depressive, anxious, and other negative emotional states, which in turn may contribute to the development of alexithymia traits. (48, 49) In contrast, patients with higher perceived social support may be more likely to utilize available material and informational resources, buffer the psychological and physiological stress associated with illness, and reduce negative emotional experiences. Enhanced perceived social support may also facilitate more effective emotion regulation and strengthen the willingness to communicate and express feelings, which may be associated with a lower likelihood of alexithymia (50, 51). Therefore, healthcare professionals should seek to identify and mobilize the social resources available to patients, encourage greater involvement and emotional support from family members and friends, and enhance communication with patients. Such efforts may help improve emotional awareness, alleviate negative affective responses, and ultimately reduce the severity or risk of alexithymia traits.

The findings of this study indicate that patients with COPD who exhibited higher levels of psychological resilience were more likely to belong to the moderate or low alexithymia groups. Jalilianhasanpour et al. (52) in a prospective study, identified high psychological resilience as an important protective factor against alexithymia. Psychological resilience, as a key internal resource, plays a crucial role when individuals encounter disease-related stress or traumatic events. It enables patients to mobilize intrinsic strengths, cultivate greater psychological robustness, and cope effectively with adversity (53). Consequently, patients with higher levels of psychological resilience tend to demonstrate greater tolerance for psychological stress and better adaptability to pressure. They are more capable of regulating negative emotional states, including anxiety and depressive symptoms, and are more likely to adopt active and constructive coping strategies in response to illness, ultimately exhibiting lower levels of alexithymia traits. These findings suggest that healthcare professionals may consider incorporating appropriate psychological interventions, such as cognitive therapy, group counseling, and mindfulness-based approaches. Such strategies may help enhance patients’ resilience, alleviate psychological distress, and potentially reduce alexithymia symptoms. However, the association between psychological resilience and alexithymia profiles did not remain statistically significant after FDR correction (P_FDR = 0.168 for Profile 2 vs 1). This finding should therefore be interpreted as preliminary, and the potential protective role of resilience requires confirmation in hypothesis-driven studies with larger sample sizes.

Further guided by the DSM, we also examined whether psychological resilience moderated the relationship between COPD duration and alexithymia profiles. The interaction term showed a marginal trend toward statistical significance for the comparison between the high and low alexithymia groups (P = 0.085). However, it was not statistically significant for the comparison between the moderate and low alexithymia groups (P = 0.466). The marginal finding for the comparison between the extreme groups suggests that the buffering effect of psychological resilience on prolonged COPD duration may be most relevant for patients at the highest risk of alexithymia. The nonsignificant result for the moderate alexithymia group may reflect the smaller differentiation between these two profiles or limited statistical power, particularly given the small sample size of the low alexithymia group (n = 24). Future studies with larger and more balanced samples are needed to further examine this interaction.

An important consideration is the clinical meaningfulness of the three-profile solution, given that the mean TAS-20 scores differed by only approximately three points across profiles. Although the absolute differences in total alexithymia scores were modest, LPA identifies subgroups based on response patterns across multiple alexithymia dimensions rather than on total scores alone. In addition, the profiles differed in clinically relevant characteristics, including physical activity intensity, perceived social support, and psychological resilience. These findings suggest that the three-profile solution may represent distinct psychosocial subgroups with different support needs and intervention priorities, rather than merely reflecting gradations of severity. Nevertheless, because the score differences were relatively small, the clinical utility and stability of this classification require further validation in larger, multicenter, and longitudinal studies.

## 5 Limitations

This study has several limitations. First, participants were recruited using convenience sampling from a single tertiary hospital in Mianyang, China. No formal *a priori* power calculation was conducted prior to data collection; however, the total sample size of 312 exceeds the generally recommended minimum of 200 for LPA (54) and is consistent with previous LPA studies in COPD populations (43). Nevertheless, several potential sources of selection bias should be considered. First, the single-center urban setting resulted in a sample that predominantly consisted of older, retired patients from a tertiary hospital, which may not represent younger patients with COPD, those with milder disease, individuals residing in rural areas, or those managed in community or primary care settings. Second, the use of convenience sampling—approaching available rather than randomly selected patients—may have introduced unknown selection bias. Third, the online survey format may have underrepresented older, less technologically literate, or more severely ill patients with COPD who may have experienced difficulty completing web-based questionnaires. Fourth, the low alexithymia group (Profile 3, n = 24, representing 7.69% of the sample) was small, which may have reduced statistical power and increased the risk of unstable parameter estimates in the multinomial logistic regression analysis. Consequently, findings related to Profile 3 should be interpreted with caution. Future studies should employ random or systematic sampling methods, expand to multicenter designs across different regions and care settings (including community and primary care), and provide alternative survey modes (e.g., paper-based questionnaires or telephone interviews) to enhance sample diversity and generalizability. Second, depressive and anxiety symptoms were assessed using the PHQ-2 and GAD-2, which are screening instruments rather than comprehensive diagnostic assessments. Their use as continuous predictors may have overestimated the precision of the reported associations. Future studies should employ more comprehensive diagnostic instruments (e.g., PHQ-9, GAD-7) or structured clinical interviews to confirm these findings. In addition, most questionnaire data were obtained through patient self-report, which may have introduced self-report bias. Future research should integrate objective and subjective approaches to assess health-related factors and alexithymia. Moreover, the online survey format may have introduced selection bias, as older, less technologically literate, or more severely ill patients with COPD may have been underrepresented. Future studies should consider alternative survey modes to enhance representativeness. Third, as noted by the reviewer, moderate correlations were observed among several psychosocial variables (GAD-2, PHQ-2, CD-RISC-10, and DT), with VIF values approaching 5. Although these values fall within generally accepted thresholds, we acknowledge that multicollinearity may have inflated standard errors to some extent, potentially affecting the precision of the corresponding coefficient estimates. Future studies with larger sample sizes should consider using factor analysis or composite indices (e.g., a general psychological distress score) to address this issue. Fourth, because cross-sectional designs cannot establish causal relationships between health-related factors and alexithymia, longitudinal studies are needed to further examine temporal and causal associations. Finally, the exploratory nature of this study involved multiple comparisons in the multinomial logistic regression model (36 tests in total). Although we applied the Benjamini–Hochberg FDR correction (threshold = 0.05) to control for false positives, this approach is conservative and may increase the risk of Type II errors (false negatives). Therefore, associations that did not remain statistically significant after FDR correction (e.g., COPD duration, psychological resilience, and depressive symptoms) should not be definitively dismissed; rather, they may represent smaller effects that require confirmation in larger, hypothesis-driven studies. Both unadjusted and FDR-adjusted p-values are reported in **Table 4** to enable readers to interpret the findings transparently.

## 6 Conclusion

In summary, this study adopted an individual-differences perspective and used LPA to examine the heterogeneity of alexithymia characteristics among patients with COPD. Significant heterogeneity in alexithymia was identified, with three distinct profiles: high alexithymia, moderate alexithymia, and low alexithymia. COPD duration, depressive symptoms, physical activity intensity, self-perceived social support, psychological resilience, and anxiety symptoms were associated with different alexithymia profiles among patients with COPD. Pending further validation in larger and more representative samples, these findings may support the development of accurate patient profiles to facilitate early identification of alexithymia subtypes and inform preventive interventions, thereby improving quality of life among patients with COPD.

## Data Availability

The raw data supporting the conclusions of this article will be made available by the authors, without undue reservation.
