# Supplementary material for: Latent profile analysis and associated factors of alexithymia among patients with chronic obstructive pulmonary disease
Source: Front Psychiatry. 2026 Jul 10;17:1823787. doi: 10.3389/fpsyt.2026.1823787 (PMC13396014; doi:10.3389/fpsyt.2026.1823787)
Supplement: Supplementary file 1 [file Supplementaryfile1.docx]

DATA: FILE IS "TSA.dat";

VARIABLE:

NAMES ARE Y1-Y3;

USEVARIABLES = Y1-Y3;

CLASSES = c(2);

ANALYSIS:

TYPE = MIXTURE;

OUTPUT:

TECH11 TECH14;

SAVEDATA:

FILE IS "File2class.txt";

SAVE = CPROB;
